# Discovery of Survivin Inhibitors Part 1: Screening the Harbor Branch Pure Compound Library

**DOI:** 10.3390/md19020073

**Published:** 2021-01-30

**Authors:** Esther A. Guzmán, Tara P. Pitts, Kirstie R. Tandberg, Priscilla L. Winder, Amy E. Wright

**Affiliations:** Harbor Branch Oceanographic Institute, Florida Atlantic University, 5600 US Highway 1, Fort Pierce, FL 34946, USA; tpitts3@fau.edu (T.P.P.); ktandberg2013@fau.edu (K.R.T.); PWINDER@fau.edu (P.L.W.); awrigh33@fau.edu (A.E.W.)

**Keywords:** survivin, eryloside E, ilicicolin H, tanzawaic acid A, high content imaging

## Abstract

Survivin is a 16.5 KDa protein whose functions include promoting cellular mitosis, angiogenesis, and senescence as well as inhibiting apoptosis. Higher survivin expression is found in cancer tissues than normal tissues, and this expression correlates with disease progression and aggressiveness. Survivin has been validated as a clinical target for cancer. Small molecules are important antagonists of survivin levels in cancer cells. A structurally diverse library of genetically encoded small molecules (natural products) derived from marine plants, invertebrates, and microbes was screened for their ability to reduce expression levels of survivin in the DLD-1 colon adenocarcinoma and the A549 nonsmall cell lung carcinoma cell lines. This led to the identification of this novel activity for the known compounds eryloside E, ilicicolin H, tanzawaic acid A, and *p*-hydroxyphenopyrrozin. Both eryloside E and ilicicolin H showed the ability to reduce survivin expression in the low micromolar range against both cell lines.

## 1. Introduction

Survivin is a 16.5 kDa protein with multiple key cellular functions [1]. Survivin was initially identified as an inhibitor of apoptosis protein (IAP) based upon the presence of a Baculovirus IAP Repeat (BIR) domain coupled to other features common in IAPs. It does not directly inhibit caspases, but is thought to interact with other antiapoptotic proteins such as X-IAP or HBXIP or sequester Smac/DIABLO to block apoptosis [2]. Survivin has been shown to be an essential participant in mitosis. It is one of four integral proteins in the chromosomal passenger complex (CPC), which is a key regulator of chromosomal segregation and cytokinesis [3]. Survivin directly binds Borealin and the inner centromere protein (INCENP) in the CPC directing chromosomal alignment, spindle formation, spindle stability and kinetochore-microtubule attachment. Survivin binds to microtubules modifying tubulin dynamics and stabilizing microtubules [4,5]. Another role of survivin is in the cellular stress response and DNA damage response (DDR) [6]. It also plays a related role in cellular senescence [2].

When the cellular functions of survivin occur in cancer cells, they facilitate cancer progression and metastasis. While survivin has been detected in normal cells [3], its expression is highly upregulated in many transformed cells suggesting a role in the pathology of these cells. Levels of survivin have been positively correlated with chemotherapy resistance, increased tumor recurrence and poor prognosis in a variety of cancers including colon, lung, and breast cancers [2,3,7,8]. Survivin expression is upregulated in tumor vascular endothelial cells and promotes angiogenesis through stimulating these cells to express and secrete vascular endothelial growth factor (VEGF), a signaling molecule essential for angiogenesis [2]. Altogether, survivin plays multiple essential roles related to cancer cell survival, proliferation, treatment resistance, and angiogenesis. The role of survivin in cancer continues to evolve even after two decades of research [1,9].

Excellent reviews are available that describe clinical targeting of survivin [10,11,12]. Molecular approaches include delivery of antisense oligonucleotides that suppress survivin mRNA, use of a dominant-negative survivin in which single amino acids at active sites are replaced, or the use of siRNA and hammerhead ribozymes to suppress survivin levels. These agents all showed favorable toxicity profiles in Phase I trials validating survivin as a tumor-selective target. Clinical trials using a vaccination approach have also shown very promising results with additional on-going trials [10]. SurVAxM is a peptide mimic, which has been evaluated against glioma in numerous trials showing encouraging efficacy and immunogenicity. The vaccine Survivin-2B-80-88 has shown promise alone and in combination therapy against a number of cancers. Small molecules are also important antagonists of survivin levels in cancer cells and have been reviewed in depth previously [10,12]. YM155 suppresses the survivin gene promoter and showed promising results in Phase I trials but later failed in Phase II trials [13]. It is a substrate for the P-glycoprotein pump (PGP) and had pharmacokinetic issues of instability and rapid clearance from blood and tumor tissue. Once cleared, it is proposed that rapid reversal of the effects of survivin downregulation occurs leading to clinical failure [10]. Newer studies where YM155 is used in combination therapy are showing more promising results in non-Hodgkin lymphoma [14]. Shepherdin is a rationally designed peptidomimetic agent that blocks binding between survivin and heat shock protein 90 (Hsp90) targeting survivin for degradation. Substantial effort was made in advancing shepherdin towards clinical trials but they have not yet taken place. FL118 is a promising survivin inhibitor, which inhibits survivin promoter activity, survivin expression, and cancer cell growth. It works through multiple mechanisms and is expected to enter clinical trials in the near future [10]. Identification of additional antagonists of survivin may help address remaining questions on the biology of survivin as well as provide entirely new therapeutic options.

The results of screening a structurally diverse library of genetically encoded small molecules (natural products) derived from marine plants, invertebrates, and microbes for their ability to reduce expression levels of survivin in the DLD-1 colon adenocarcinoma and the A549 nonsmall cell lung carcinoma cell lines are described herein. The A549 and DLD-1 cell lines used in this study contain activating mutations in the Ras oncogene. When mutated, Ras can accelerate tumor initiation and progression [15,16]. About 30% of all cancers are driven by Ras mutations. It is estimated that 95% of pancreatic cancers, 45% of colorectal, 35% of lung cancers, and 15% of melanomas are driven by Ras mutations [17]. Activating mutations in Ras have been shown to upregulate levels of survivin [18,19]. Knockdown of survivin levels using small interfering RNA (siRNA) significantly reduced the survival of activated K-Ras-transformed cells compared with a normal isogenic counterpart in which the mutant K-Ras gene had been disrupted [20]. Thus, it is expected that compounds identified through this effort would have the potential to be useful against a variety of cancers bearing this mutation.

## 2. Results

To identify novel modulators of survivin levels (inhibitors) among our collection of marine-derived secondary metabolites, a cell-based high content imaging screening assay was established. Both the DLD-1 and the A549 cell lines have been reported to express high levels of survivin [20,21]. The known inhibitors of survivin YM155 [22,23] and oxaliplatin [24] were used as positive controls. For the assay, cells were treated with marine-derived compounds or controls for 24 h and then labeled with fluorescent survivin antibody, a nucleic acid stain, and a cell mask. Images were acquired with the high content imager and analyzed to determine survivin expression levels and DNA content to measure cytotoxicity. Samples were scored in two ways. The first method designated *survivin expression* represents the change in percentage of cells scored positive for the presence of survivin overall fluorescence in treated cells versus control cells. Survivin overall fluorescence may be the result of one or more antibodies specific to survivin binding to a single cell; thus, this is a measurement of the change in the number of cells that exhibit fluorescence due to the presence of survivin. The second method designated as the *fluorescent intensity* is dependent on the integrated intensity of the fluorescent signal from specific binding of fluorescent antibodies to survivin per cell [25] and measures the average fluorescent intensity of all cells. Cells with multiple bound antibodies will have higher fluorescent intensity than cells where fewer antibodies have bound.

**Figure 1:**
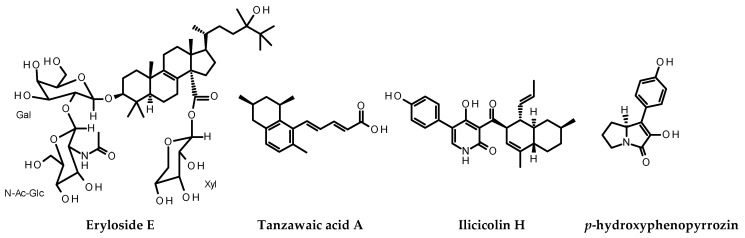
Structures of marine natural products identified to have the novel activity of decreasing survivin levels in cancer cells.

**Figure 2:**
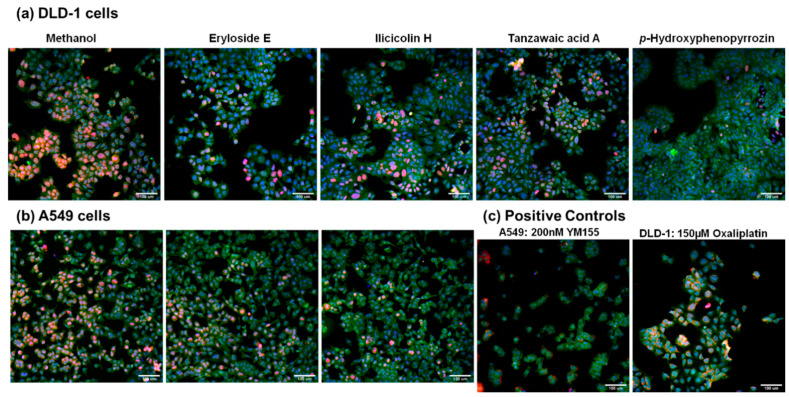
Downregulation of survivin detected by immunofluorescence. A high content imaging assay was set up to measure effects on the levels of survivin in the A549 and DLD-1 cell lines after treatment with 5 µg/mL marine samples for 24 h. Levels were normalized to their respective solvent controls for ease of comparison. Samples showing ≥50% reduction in survivin expression and ≤20% cytotoxicity were considered hits. Nine sites were imaged per well. One representative image for each compound is shown. Survivin: red; Hoechst 33342 nuclear stain: blue; cytoplasm stain: green. (**a**) Images for the DLD-1 human colorectal adenocarcinoma cell line (**b**) images for the A549 human lung carcinoma cell line. (**c**) Known inhibitors of survivin were used as positive controls at a single concentration based on previously reported activity in the literature: 200 nM YM155 was used as the positive control for A549 cells, while 150 µM oxaliplatin was used as the positive control for DLD-1 cells. Screening results were confirmed by repetition.

Samples that showed ≥50% reduction in survivin expression and exhibited ≤20% cytotoxicity at a concentration of 5 μg/mL were considered hits. Samples that showed ≥50% reduction in survivin fluorescent intensity with ≤20% cytotoxicity at a concentration of 5 μg/mL were also considered hits. This led to the identification of the novel activity of reducing survivin levels in cancer cells for the known compounds eryloside E, ilicicolin H, tanzawaic acid A, and *p*-hydroxyphenopyrrozin whose structures are shown in Figure 1. Representative images from one experiment in the screening assay are shown in Figure 2.

**Figure 3:**
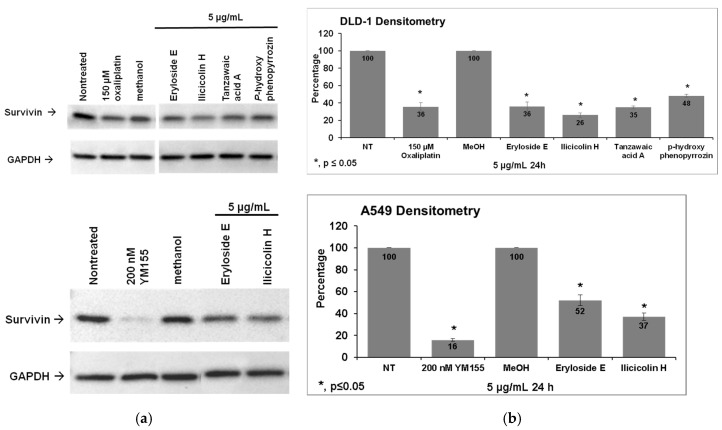
Confirmation of survivin inhibition by Western blotting. (**a**) The expression of survivin in DLD-1 and A549 cells treated with 5 μg/mL marine samples or controls for 24 h was ascertained using Western blotting. The ability of the compounds to reduce survivin expression in DLD-1 and A549 cells was confirmed. Western blot for one representative experiment is shown. (**b**) Densitometry analysis showed that this decrease was significant (*p* < 0.05) in samples marked with an asterisk. Graph shows the average densitometry ± standard deviation for three experiments.

To confirm the decrease in survivin expression observed in the primary screen, the expression levels of survivin in DLD-1 and A549 cells treated for 24 h with 5 μg/mL marine compounds or controls was determined using Western blotting. As shown in Figure 3, the ability of the compounds to reduce expression levels of survivin was confirmed in the cell line in which the compound was identified as a hit.

After confirmation of reduction in survivin expression by Western blotting, the effective concentration that reduced survivin fluorescence expression levels by 50% was determined (EC_50_). As described in the methods, serial dilutions ranging from 20 to 0.04 μg/mL marine compounds were tested in the screening assay. The expression levels were normalized to methanol (vehicle control) and the values expressed as a percentage were subjected to a nonlinear regression curve fit analysis. The graphs from this analysis are shown in Supplementary Materials in Figure S9. The calculated values are shown in Table 1. Molar concentrations are provided to better compare the potency of the different compounds. As shown in Table 1, the most potent compounds were eryloside E and ilicicolin H.

Reduction in fluorescent intensity was also used as a parameter to select hits. Integrated fluorescent intensity gives an idea of the quantity of antibodies binding each cell. The concentration required to see a 50% reduction in survivin fluorescent intensity was calculated using serial dilutions ranging from 20 to 0.04 μg/mL of marine compounds tested using the screening assay. The fluorescent intensity levels were normalized to methanol (vehicle control) and subjected to a nonlinear regression curve fit analysis. The graphs from this analysis are shown in the Supplementary Materials Figure S10. The resulting values are shown in Table 2. The most potent compound was eryloside E, followed by ilicicolin H. Both tanzawaic acid A and *p*-hydroxyphenopyrrozin are more potent in reducing survivin fluorescent intensity than in reducing survivin expression.

**Figure 4:**
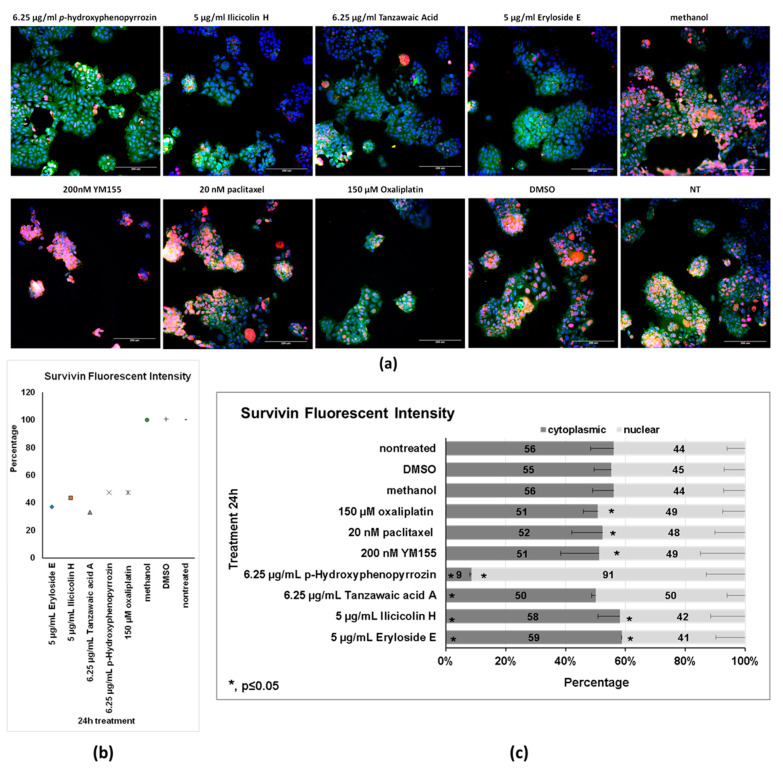
Effects of the compounds on survivin fluorescent intensity in DLD-1 cells. Fluorescent Intensity gives an idea of the quantity of antibodies binding each cell. This analysis used data from the EC_50_ measurements at the concentration closest to the original screening concentration of 5 µg/mL (6.25 µg/mL for *p*-hydroxyphenopyrrozin and tanzawaic acid A and 5 µg/mL for ilicicolin H and eryloside E). (**a**) Representative Images from a single experiment. (**b**) Overall fluorescent intensity. All of the compounds tested had the ability to decrease fluorescent intensity. Data shown are the average of three independent experiments. (**c**) Localization. High content analysis allowed for the determination of whether the expression was nuclear or cytoplasmic and how this was affected by treatment. Most compounds increased the amount of nuclear survivin, with *p*-hydroxyphenopyrrozin causing the most dramatic shift. Data shown is the average ± percent error of three independent experiments.

The reduction in fluorescent intensity in the DLD-1 cells was looked at in more detail at a single concentration using the data generated to determine the EC_50_ (shown in Table 2). The data for integrated fluorescence intensity, cytoplasmic integrated intensity and nuclear integrated intensity from three independent experiments at the concentration closest to the original screening concentration of 5 µg/mL (6.25 µg/mL for *p*-hydoxyphenopyrrozin and tanzawaic acid A and 5 µg/mL for ilicicolin H and eryloside E) were used for this more detailed look. As shown in Figure 4a, all compounds reduced survivin fluorescent intensity to levels similar or greater than oxaliplatin, a known inhibitor of survivin used as the positive control in this cell line. Both YM155 and paclitaxel showed a significant increase in fluorescent intensity that was ascribed to their ability to induce cell cycle arrest (data not shown). Nuclear versus cytoplasmic localization of survivin in tumor tissues has been reported to correlate with patient prognosis both positively and negatively dependent upon tumor type [26]. Therefore, the effects of treatment on cellular localization of survivin fluorescent intensity was also determined and is shown in Figure 4b. The localization of survivin for the solvent controls matched that seen in the nontreated cells, showing very similar percentages of cytoplasmic and nuclear survivin fluorescent intensity with the cytoplasmic slightly favored. The drug controls paclitaxel, YM155, and oxaliplatin, showed small increases in the amount of nuclear survivin in a statistically significant manner (*p* ≤ 0.05). The decrease in cytoplasmic survivin caused by YM155 and paclitaxel failed to be statistically significant, probably because there was more variability in the data (see error bars). *p*-Hydroxyphenopyrrozin increased the amount of nuclear survivin in a dramatic and statistically significant manner with 91% of the remaining survivin being localized in the nucleus. This could be due to a number of reasons including degradation of cytoplasmic survivin or block of export of nuclear survivin. Further work is required to determine how this occurs or its relevance to cancer treatment. Tanzawaic acid A showed a slight increase of nuclear survivin that failed to be statistically significant. The other compounds caused very slight changes, albeit statistically significant, compared to solvent controls. These changes may not be large enough to have functional relevance.

Samples were selected as hits because they showed little cytotoxicity in the screening assay. Nevertheless, an attempt was done to determine the IC_50_ for cytotoxicity in the DLD-1 cell line. As shown in Table 3, for all compounds, the IC_50_ was not achieved and it was determined to be greater than 20 µg/mL.

**Figure 5:**
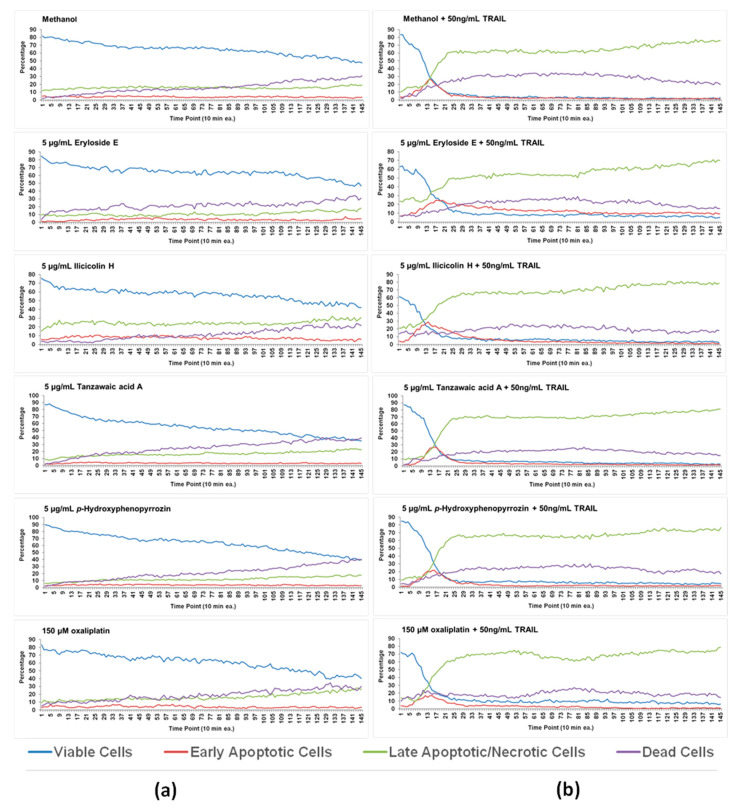
Effects of decrease in survivin expression on apoptosis. DLD-1 cells were plated, allowed to adhere overnight, then treated for 24 h with 5 μg/mL marine compounds or methanol (control) in the presence or absence of 50 ng/mL killer TRAIL. Immediately after treatment, cells were stained with the cell permeable nuclear stain Hoechst 33342, the cell impermeable nucleic stain 7-aminoactinomycin D (7AAD) and a fluorescent substrate for caspase 3/7. Cells were imaged every 10 min for 24 h. Four sites were imaged per well. Graphs shown represent the average of all fields. At least three independent experiments were performed. One representative experiment is shown. (**a**) shows the effects of compounds and controls without killer TRAIL and (**b**) shows the effects of compounds and controls with killer TRAIL.

Given the role of survivin in inhibiting apoptosis there was the possibility that the compounds could sensitize the cells to undergo apoptosis. Therefore, the ability of the compounds to induce apoptosis or sensitize cells to undergo tumor necrosis factor-related apoptosis-inducing ligand (TRAIL) induced apoptosis. Many chemotherapies sensitize cells to undergo TRAIL-induced apoptosis [27]. As shown in Figure 5, none of the compounds induced apoptosis in the DLD-1 cells on their own. DLD-1 cells seemed to be rather susceptible to apoptosis induced by recombinant TRAIL and none of the compounds tested seemed to further sensitize cells to undergo TRAIL-induced apoptosis.

## 3. Discussion

Screening the HBOI collection of marine-derived natural products has led to the identification of compounds that have the ability to reduce survivin levels in cancer cells. Survivin is an important regulator of apoptosis, cell cycle, and DNA repair pathways with preferential expression in cancer tissue over normal tissues. The activities regulated by survivin can facilitate tumor initiation and progression. Therefore, the activity of reducing survivin expression by these marine natural compounds gives them the potential to be chemotherapeutic for lung or colon cancer.

As with all phenotypic screening, future experiments are needed to determine the mechanism by which these compounds cause this effect. Some of these compounds were active against both cell lines which is not always the case with survivin inhibitors/modulators. For example, YM155 did not reduce survivin levels in DLD-1 cells. While these compounds were more potent than oxaliplatin, they were not as potent as YM155. In this screening, we selected noncytotoxic compounds as hits to avoid false negative so the lack of effects on apoptosis were not completely unexpected. However, the testing was not exhaustive. Apoptosis was ascertained at the same time when the reduction in survivin was determined and perhaps a longer incubation period is necessary to see effects. The cells were very susceptible to TRAIL-induced apoptosis and thus using a lower concentration of recombinant TRAIL would better allow to ascertain the ability of the compound to sensitize cells to undergo apoptosis. Finally, survivin is known to regulate intrinsic apoptosis. While inducers of extrinsic apoptosis such as TRAIL can trigger intrinsic apoptosis in some cells, using a different inducer that activates intrinsic apoptosis may be a better approach to determine the ability of the compounds to sensitize cancer cells to apoptosis. Performing these in-depth experiments as well as experiments to obtain insights on the mechanism of these compounds will be necessary to determine how these compounds compare to known survivin inhibitors/modulators.

The compounds highlighted here are not new structures, but their ability to reduce survivin expression in cancer cells is novel for them. Many had a few, if any, activities against cancer previously reported.

Eryloside E is a terpene glycoside that was first isolated from the sponge *Erylus goffrilleri* collected in the Bahamas. It was reported to inhibit binding of ^125^[I]-Bolton Hunter labelled C5a to its receptor (IC_50_ > 10 µM). It also showed immunosuppressive activity in the mixed lymphocyte reaction assay with an EC_50_ of 1.3 µg/mL and a TC_50_ of 12.3 µg/mL in a lymphocyte viability assay [28].

Ilicicolin H was first isolated from the mycelium of the fungus *Cylindrocladium ilicicola* and was reported to have antibiotic activity against *Bacillus anthracis* and moderate cytotoxicity against HeLa cervical cancer cells (IC_50_ 4.6 µM) [29]. Its structure was defined a few years later [30]. Ilicicolin H inhibits the yeast cytochrome bc1 complex [31] and has broad antifungal activity [32].

Tanzawaic acid A was first isolated from *Penicillium citrinum* [33]. It has been reported to have anti-inflammatory activity [34,35] and to inhibit the activity of protein tyrosine phosphatase 1B (PTP1B) [35]. PTP1B regulates many signaling cascades that promote tumor progression and survival and is considered a therapeutic target against cancer [36]. PTP1B inhibitors have been shown to cause cell cycle arrest and inhibit the protein expression of survivin and other regulators of apoptosis and cell cycle progression in Hep G2 liver cancer cells [36]. This known mechanism of action thus may explain its ability to reduce survivin levels. Tanzawaic acid was chosen as a hit because of its ability to reduce overall fluorescent intensity.

*p*-Hydroxyphenopyrrozin, or tetrahydropyrrolizin-3-one-5,6,7,7a-2OH-1-(p-OH)phenyl, was first isolated from the marine-derived fungus *Chromocleista* sp. found in a deep-water sediment sample collected in the Gulf of Mexico [37]. *p*-Hydroxyphenopyrrozin showed a minimum inhibition concentration of 25 µg/mL (108 µM) against *Candida albicans*, and no activity against *Staphylococcus aureus* [37]. No cytotoxicity for this compound was detected when tested at 5 µg/mL (22 µM) against the cancer cell lines P388 (murine leukemia), A549 (human lung adenocarcinoma), PANC-1 (human pancreatic carcinoma), and NCI/ADR-RES (human ovarian cancer) [37]. The structurally related compound phenopyrrozin was isolated from a fungus and shows antimicrobial and radical scavenger activity [38]. While *p*-hydroxyphenopyrrozin was not the most potent compound, it did appear to affect the cellular localization of survivin when fluorescent intensity was looked at in more detail. The localization of survivin may have profound effects on function, as most of its regulation of apoptosis occurs in the cytoplasm while the regulation of cell cycle occurs at the nucleus.

In all cases where the compounds showed activity in both cell lines, compounds were more potent against DLD-1 cells than against A549 cells. A549 cells are p53 wild-type while DLD-1 cells have p53 mutations [39]. Wild type p53 represses survivin expression [40] and this may be one reason why a higher concentration was needed to see survivin downregulation in cells where the survivin promoter is already inhibited. Both the A549 and DLD-1 were chosen as they contain Ras mutations that are associated with increase in survivin, which contributes to aggressiveness of tumors. Compounds which reduce the levels of survivin have been shown to block the proliferation of cells with activating mutations in Ras and may be useful against Ras-driven cancers. Both eryloside E and ilicicolin H showed reduction in survivin levels in the low micromolar range against both cell lines and further work could delineate their utility against these cancers.

## 4. Materials and Methods

### 4.1. Reagents

Compounds used in this study were obtained from the Harbor Branch Oceanographic Institute Pure Compound Library at a concentration of 1 mg/mL in methanol. Methanol used in the experiments was purchased from Fisher Scientific, Fair Lawn, NJ, USA. The identity and purity of all compounds was confirmed by high-performance liquid chromatography (HPLC) with photodiode array and evaporative light scattering detection (Supplementary Figures S1–S4), high-resolution mass spectrometry (Supplementary Figures S5–S8) and NMR.

### 4.2. Cell Culture

The human colon adenocarcinoma DLD-1 (CCL-221) and non-small cell lung carcinoma A549 (CCL-185) cell lines were obtained from ATCC (Manassas, VA, USA) and grown, aliquoted, and maintained in liquid nitrogen. The A549 cell line was grown in RPMI-1640 supplemented with 10% fetal bovine serum (HyClone Laboratories Inc., Logan, UT, USA), 18 mM HEPES buffer, 100 U/mL penicillin G sodium, 100 μg/mL streptomycin sulfate, 0.25 μg/mL amphotericin B, 2 mM l-glutamine, and 50 μg/mL gentamicin. DLD-1 were grown in the same media that was further supplemented with 0.11 mg/mL sodium pyruvate and 4.5 g/L d-glucose. Media was purchased from ATCC and supplements from Gibco (Thermo Fisher, Waltham, MA, USA). Cells were maintained in a humidified incubator at 37 °C and 5% CO_2_. Cells were kept in culture for 10 weeks (20 passages) when a new aliquot was thawed.

### 4.3. Survivin High Content Imaging Screening Assay

Precisely, 3500 A549 or 4000 DLD-1 cells/well were plated on 384-well black plates with clear bottom (Greiner #781097, Greiner Bio-One, Monroe, NC, USA) and allowed to adhere overnight. A dilution plate was prepared containing 5 μg/mL marine compounds, 200 nM YM155 (A549 positive control), 150 μM oxaliplatin (DLD-1 positive control), 50 nM Taxol (A549) or 20 nM Taxol (DLD-1), respective solvent controls, and nontreated media alone (NT). Media was removed by aspiration and replaced with media containing treatments. After a 24 h incubation, media was removed and cells were fixed with 4% paraformaldehyde followed by permeabilization with ice cold methanol. Antibody staining happened on the day images were acquired. Cells were blocked with blocking buffer (1X PBS/5% normal serum/0.3% Triton X-100) for 1 h at room temperature and then labeled with survivin antibody (Cell Signaling Technology, Danvers, MA; clone (6E4), Cat #2802) diluted 1:100 in Antibody Dilution Buffer (1XPBS/1% BSA/0.3% Triton X-100). This was followed by labeling with secondary antibody donkey anti-mouse IgG Alexa Fluor 647 (Jackson ImmunoResearch, West Grove, PA, USA; Cat# 715-606-151) diluted 1:300 in dilution buffer containing green cell mask (Molecular Probes, Eugene, OR, USA; Cat # H32714) and Hoechst 33342 (Molecular Probes, Cat # R37605). After antibody labeling, cells were washed with PBS and then wells were filled with distilled water. Images from 9 sites per well were captured with an ImageXpress Micro XLS High Content Imager (Molecular Devices, San Jose, CA, USA) using a 20X objective and resulting images were analyzed using the multi-wavelength cell scoring module of MetaXpress 5.3.0.5 Software (Molecular Devices). Data were normalized to vehicle control and plotted using Excel (Microsoft, Redmond, WA, USA). Samples were scored in two ways: (1) Reduction in survivin expression represents the change in percentage of cells scored positive for survivin overall fluorescence in treated cells versus control cells. Survivin overall fluorescence may be the result of a single antibody or multiple antibodies specific to survivin binding to a single cell, thus this is a measurement of the number of cells that exhibit fluorescence due to the presence of survivin. (2) Integrated fluorescent intensity is dependent on the number of fluorescent antibodies that specifically bind to a single cell. Cells with multiple antibodies binding will have higher fluorescent intensity than cells where fewer antibodies bound. Fractions that decreased survivin expression levels by 50% or more with less than 20% cytotoxicity were considered hits. Cytotoxicity was determined based on DNA content. Samples were tested in duplicate within a plate. Activity was confirmed by repeating the testing.

### 4.4. EC_50_ Calculation

The concentration of the compound required to obtain 50% reduction in survivin expression levels (EC_50_) was calculated by testing serial dilutions from the samples ranging from 20 to 0.04 μg/mL marine compounds in the above assay. The resulting expression levels were normalized to methanol (vehicle control), and the values were subjected to a nonlinear regression curve fit with GraphPad Prism 5 software (La Jolla, CA, USA). The same approach was followed to calculate the concentration at which 50% reduction in survivin fluorescent intensity is seen.

### 4.5. Western Blotting

Cells were seeded at normal density, allowed to adhere overnight, and then treated with 5 µg/mL marine samples or methanol for 24 h. At the end of the incubation, media and trypsinized cells were pooled and pelleted by centrifugation. Cells were lysed in lysis buffer (10 mM Tris-Cl pH 7.5, 100 mM NaCl, 0.5% Nonidet P-40, 1 mM phenylmethylsulfonyl fluoride, Halt Protease Inhibitor Cocktail (Pierce, Rockford, IL), 1 mM Na_3_VO_4_, 1 mM NaF) for 30 min at 4 °C, followed by centrifugation to pellet the cell debris. The supernatant containing the protein was transferred to a new tube, quantitated using BCA Protein Assay Kit (Pierce, Rockford, IL, USA) and stored at –80 °C. Protein (20 µg) was run in a precast, 4–20% SDS-PAGE gel (Bio-Rad, Hercules, CA, USA), which was then transferred to a polyvinylidene difluoride (PVDF) membrane (Bio-Rad, Hercules, CA, USA), and blocked with 5% nonfat milk in Tris-buffered saline containing Tween-20 (TBST) buffer for 1 h at room temperature. After repeated washing, the membrane was incubated with primary antibody survivin (#2802) diluted 1:1000 or GAPDH (#2118) diluted 1:4000; both obtained from Cell Signaling Technology, Danvers, MA, USA followed by peroxidase conjugated secondary antibody (respectively) goat anti-mouse IgG (#115-035-146) diluted 1:10,000 or donkey anti-rabbit IgG (#711-035-152) diluted 1:200,000 both obtained from Jackson ImmunoResearch, West Grove, PA, USA, followed by repeated washing. Detection of proteins was done with chemiluminescence (Bio-Rad, Hercules, CA, USA), followed by imaging with the ChemiDoc MP System and densitometry analysis using Image Lab 4.1 software (Bio-Rad, Hercules, CA, USA). Western blots were repeated 3 times.

### 4.6. Live Cell Apoptosis Assay

A total of 4500 cells/well were plated in a 384 black clear bottom plate (Greiner 781097) and allowed to adhere overnight. The environmental chamber of the ImageXpress Micro XLS High Content Imager (Molecular Devices, San Jose, CA, USA) was set up to a temperature to 37 °C and 5% CO_2_ level an hour before treating cells. When chamber was ready, media was removed by aspiration and replaced by fresh media (complete RPMI with no phenol red) containing treatments. Treatments consisted of a final concentration of 5 µg/mL marine samples, solvent controls, and media alone or 200 nM YM155 (A549 cells) or 150 µM oxaliplatin (DLD-1 cells) in the presence or absence of 50 ng/mL recombinant killer TRAIL (Alexis Biochemicals now Enzo Life Sciences, Farmingdale, NY, USA). In addition to treatment, a staining mixture consisting of 0.25 μg 7-Aminoactinomycin D (7-AAD; Sigma A9400), 5 μM CellEvent™ Caspase-3/7 Green Detection Reagent (Molecular Probes C10423), and 2 drops/1000 mL NucBlue Live Cell Stain Hoechst 33342 (Molecular Probes R37605) was added to each well. The assay plate was placed briefly on the microtiter plate shaker to mix treatment with dye without disturbing cells. The high content imager acquired images from 4 sites/well every 10 min for 24 h under the appropriate filters for each stain at 20× magnification. The resulting images were analyzed using the cell health module of MetaXpress 5.3.0.5 Software (Molecular Devices). Data were normalized and plotted using Excel (Microsoft, Redmond, WA, USA). Images for 145 time points were overlaid, stacked, and turned into a movie using MetaXpress 5.3.0.5 Software. The speed was optimized and captions added using Microsoft Movie Maker software. The experiment was repeated at least three times.

### 4.7. Statistics

Statistical analysis of the data sets to determine mean and standard deviation was performed using Microsoft Excel. Experiments were repeated a minimum of three times. Data sets were compared using the Student’s *t* test. A *p* value ≤ 0.05 was considered significant. Outliers were detected through the Grubbs’ test.

## Figures and Tables

**Table 1:** Reduction in survivin expression.

Compound	MW	EC_50_ μg/mL		EC_50_ μM	
		DLD-1	A549	DLD-1	A549
Eryloside E	1000	6.4 ± 0.1	11 ± 0.2	6.4 ± 0.1	11 ± 0.2
Ilicicolin H	433	4 ± 0	4.1 ± 0.2	9.3 ± 0.1	9.5 ± 0.5
Tanzawaic acid A	270	17 ± 0.2		63 ± 0.7	
*p*-Hydroxyphenopyrrozin	231	5.8 ± 0.1		25 ± 0.5	

The concentration of marine compounds needed to give 50% reduction in survivin expression (EC_50_) was determined using a nonlinear regression curve fit. Reduction in survivin expression represents the change in percentage of cells scored positive for survivin overall fluorescence in treated cells versus control cells. Data shown is the average ± standard deviation of three independent experiments.

**Table 2:** Reduction in survivin integrated fluorescent intensity.

Compound	MW	EC_50_ μg/mL		EC_50_ μM	
		DLD-1	A549	DLD-1	A549
Eryloside E	1000	4.0 ± 0.3	7.1 ± 0.2	4.0 ± 0.3	7.1 ± 0.2
Ilicicolin H	433	2.8 ± 0	4.2 ± 0.2	6.5 ± 0.1	9.8 ± 0.4
Tanzawaic acid A	270	3.0 ± 0.2		11 ± 0.7	
*p*-Hydroxyphenopyrrozin	231	5.1 ± 0.1		22 ± 0.5	

The concentration needed to give 50% reduction in fluorescent intensity (EC_50_) was determined using serial dilutions ranging from 20 to 0.04 μg/mL marine compounds tested using the screening assay. The fluorescent intensity levels were normalized to methanol (vehicle control) and the values were subjected to a nonlinear regression curve fit analysis. Data shown is the average ± standard deviation of three independent experiments.

**Table 3:** IC_50_ for cytotoxicity in DLD-1 cells.

Compound	DLD-1 (IC_50_ µM) ^1^
Eryloside E	>20
Ilicicolin H	>46
Tanzawaic acid A	>74
*p*-Hydroxyphenopyrrozin	>87

^1^ The concentration of marine compounds needed to give 50% inhibition in cell viability (IC_50_) in the DLD-1 cell line was determined using a nonlinear regression curve fit.

## Data Availability

Any data not included in the manuscript or supplementary materials that supports the work presented in this manuscript is available upon reasonable request to the corresponding author (E.A.G.).

## Supplementary Materials

The following are available online at https://www.mdpi.com/1660-3397/19/2/73/s1, Figure S1. HPLC chromatogram with PDA and ELSD detection of Eryloside E; Figure S2. High-resolution ESI positive ion mass spectrum of Eryloside E used in the study; Figure S3. HPLC chromatogram with PDA and ELSD detection of Ilicicolin H; Figure S4. High-resolution DART positive ion Mass spectrum of Ilicicolin H used in the study; Figure S5. HPLC chromatogram with PDA and ELSD detection of tanzawaic acid A; Figure S6. High-resolution DART positive ion Mass spectrum of tanzawaic acid A used in the study; Figure S7. HPLC chromatogram with PDA and ELSD detection of *p*-hydroxyphenopyrrozin; Figure S8. High-resolution DART Mass spectrum of *p*-hydroxyphenopyrrozin used in the study. Figure S9. EC_50_ graphs for the reduction in survivin expression. Figure S10. EC_50_ graphs for the reduction in survivin fluorescent intensity. Figure S11. Larger pictures for Figure 1. Figure S12. Larger pictures for Figure 4.
