# Supplementary material for: Discovery of Survivin Inhibitors Part 1: Screening the Harbor Branch Pure Compound Library
Source: Mar Drugs. 2021 Jan 30;19(2):73. doi: 10.3390/md19020073 (PMC7911841; doi:10.3390/md19020073)
Supplement: Supplementary file 1 [file marinedrugs-19-00073-s001.pdf]

Supplementary Materials

Discovery of Survivin Inhibitors Part 1: Screening the Harbor Branch Pure Compound Library

Esther A. Guzmán*, Tara P. Pitts, Kirstie R. Tandberg, Priscilla L. Winder, Amy E. Wright

Harbor Branch Oceanographic Institute, Florida Atlantic University, 5600 US Highway 1, Fort Pierce, FL 34946, USA

Figure S1. HPLC chromatogram with PDA and ELSD detection of Eryloside E

Figure S2. High resolution ESI positive ion mass spectrum of Eryloside E used in the study

Figure S3. HPLC chromatogram with PDA and ELSD detection of Ilicicolin H

Figure S4. High resolution DART positive ion Mass spectrum of Ilicicolin H used in the study

Figure S5. HPLC chromatogram with PDA and ELSD detection of Tanzawaic Acid A

Figure S6. High resolution DART positive ion Mass spectrum of Tanzawaic Acid A used in the study

Figure S7. HPLC chromatogram with PDA and ELSD detection of *p*-hydroxyphenopyrrozin

Figure S8. High resolution DART Mass spectrum of *p*-hydroxyphenopyrrozin used in the study

Figure S9. EC_{50} Graphs for the Reduction in Survivin Expression.

Figure S10. EC_{50} Graphs for the Reduction in Survivin Fluorescent Intensity.

Figure S11. Larger pictures for Figure 1.

Figure S12. Larger pictures for Figure 4.

Figure S1. HPLC chromatogram with PDA and ELSD detection of Eryloside E

D-2000: JennSandle2017-2 Series: 0259 Report: original System: HPLC 1
018

D-2000 Elite HPLC System Manager Report

Analyzed: 09/13/2018 10:23 AM Reported: 09/13/2018 10:59 AM
Sample Name: HB-214
Sample Description: 1mg/ml

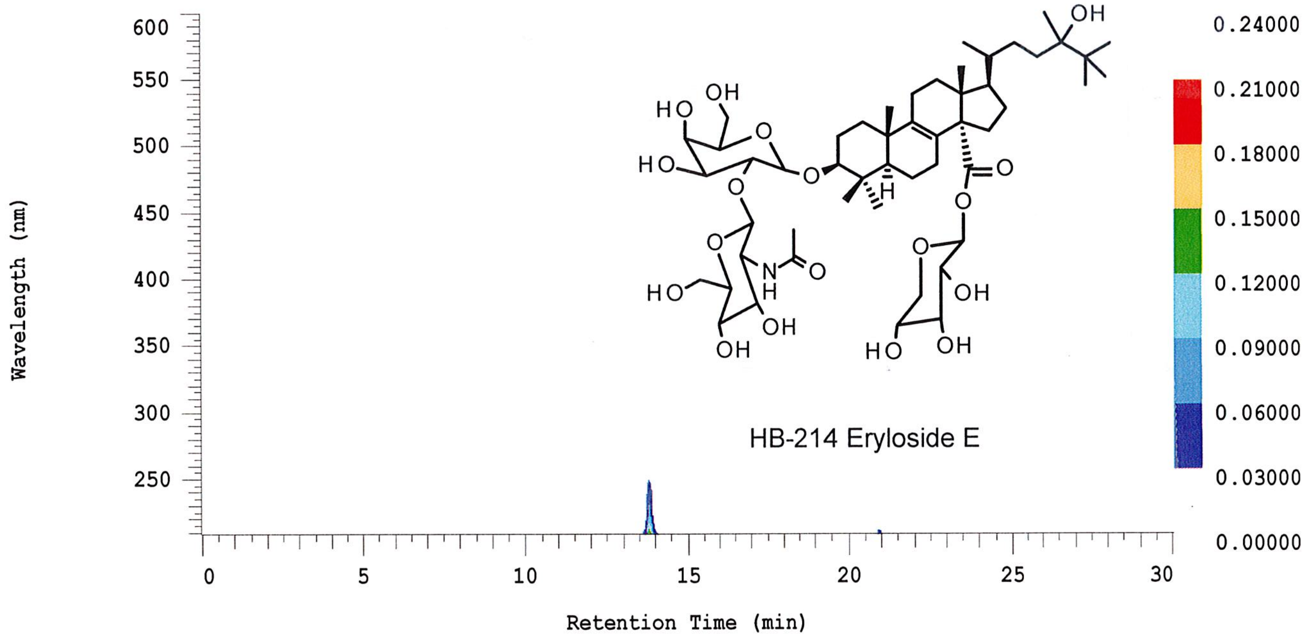

Channel 1 Chrom Type: Fixed WL Chromatogram, 230 nm

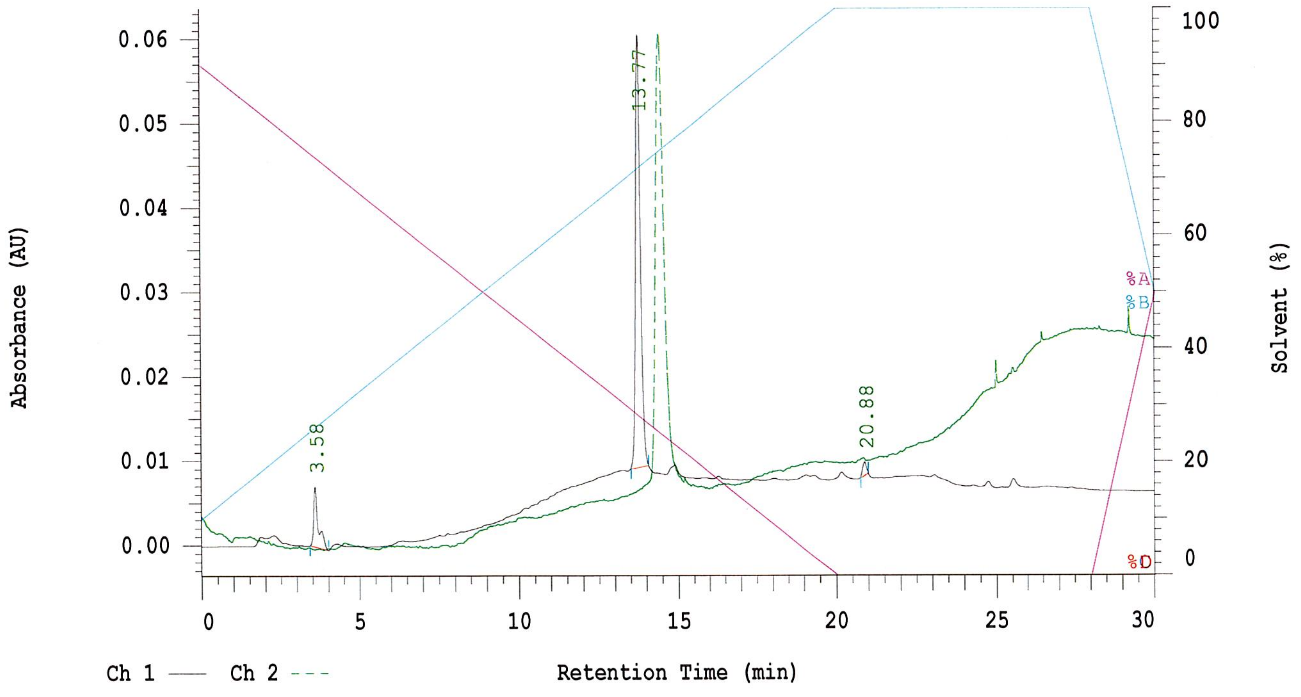

Acquisition Method: NOautosampler_30min_wELSD
Column Type: Vydac C18
Pump A Solvent A: H2O/5% ACN Pump A Solvent B: ACN
Method Description:

Figure S2. High resolution ESI positive ion mass spectrum of Eryloside E used in the study

Elemental Compositions

D:\msAxel@LP Data\Amy data\Samples\HB-214_ErylosideE_ESI+.txt

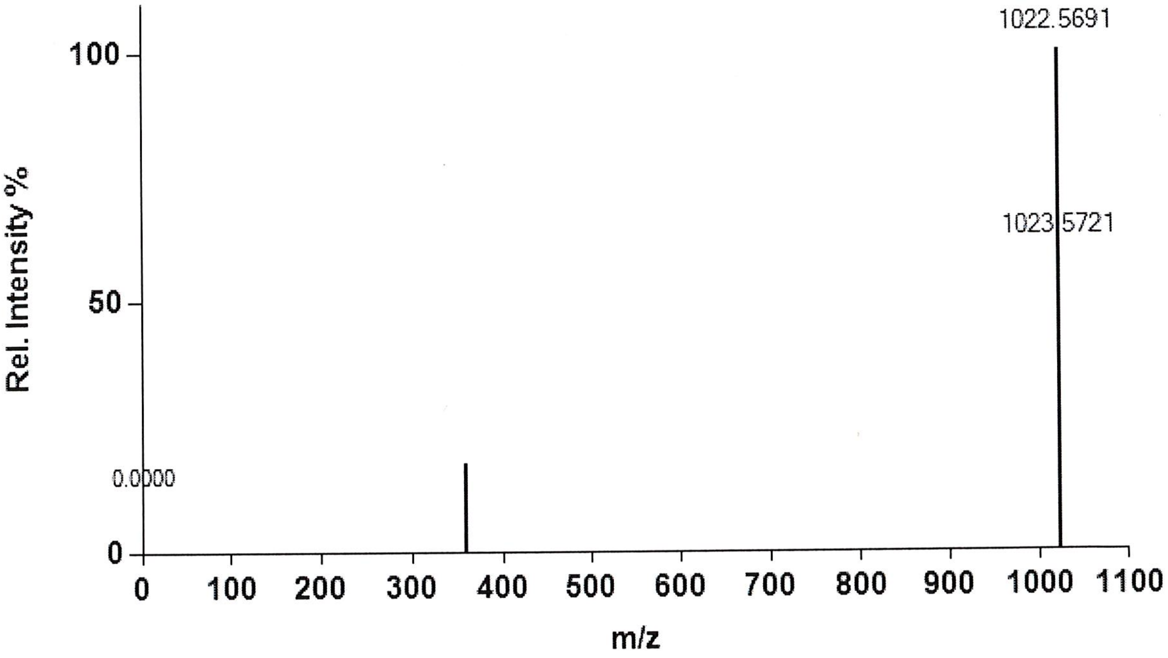

Mass Spectrum

Elemental Compositions
Element Limits: C 0/51 H 0/86 O 0/20 N 0/1 Na 0/1
Tolerance: 10 mmuEven or odd electron ion or both: Even
Electron correction: None.Charges: 1
Minimum unsaturation: -1Maximum unsaturation: 100

Calc. m/z	Abund %	mmu	DBE	Composition
1022.566435	100.000	-2.66	9.5	C51H85O18N1Na1

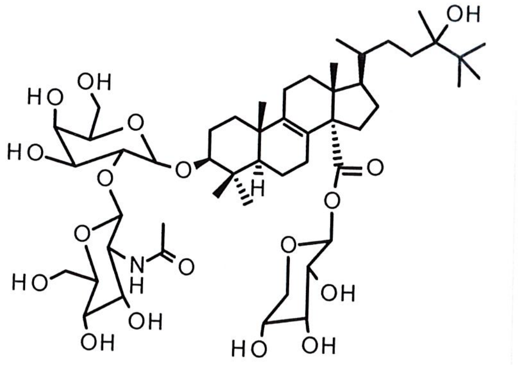

HB-214 Eryloside E

Formula Weight : 1000.22(4)
Formula : $C_{51}H_{85}NO_{18}$

Figure S3 HPLC chromatogram with PDA and ELSD detection of Ilicicolin H

D-2000: JennSandle2017-2 Series: 0260 Report: original System: HPLC 1
018

D-2000 Elite HPLC System Manager Report

Analyzed: 09/13/2018 11:01 AM   Reported: 09/13/2018 11:39 AM

Sample Name: HB-322

Sample Description: 1mg/ml

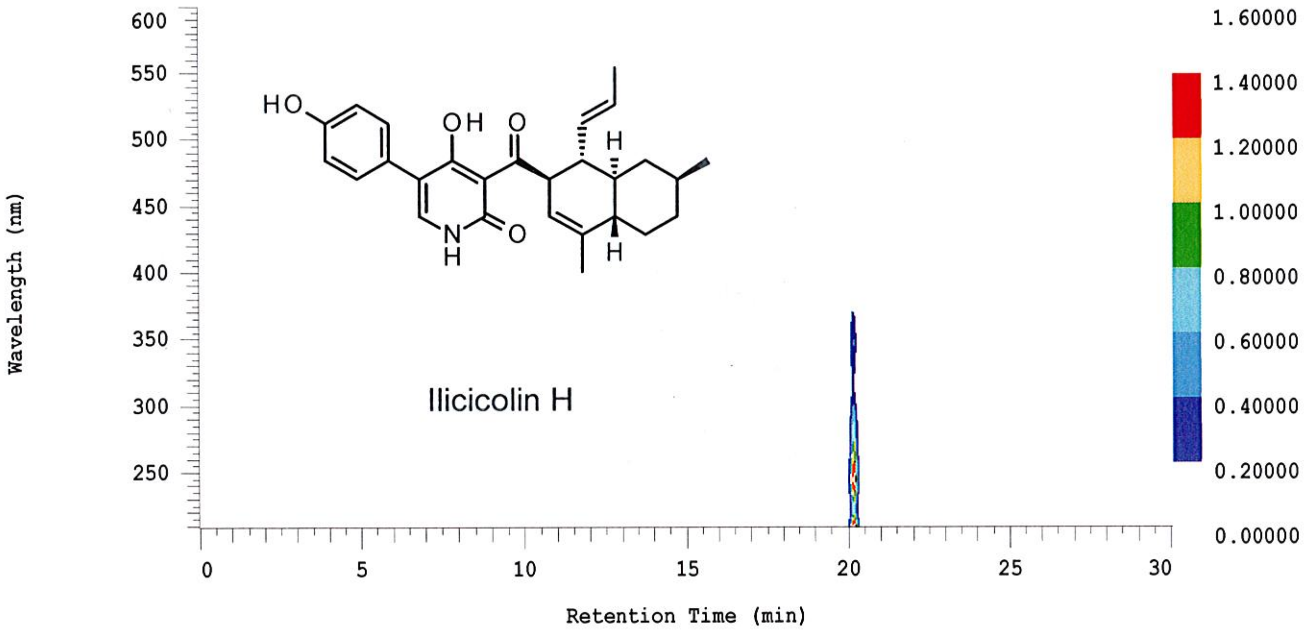

Channel 1 Chrom Type: Fixed WL Chromatogram, 230 nm

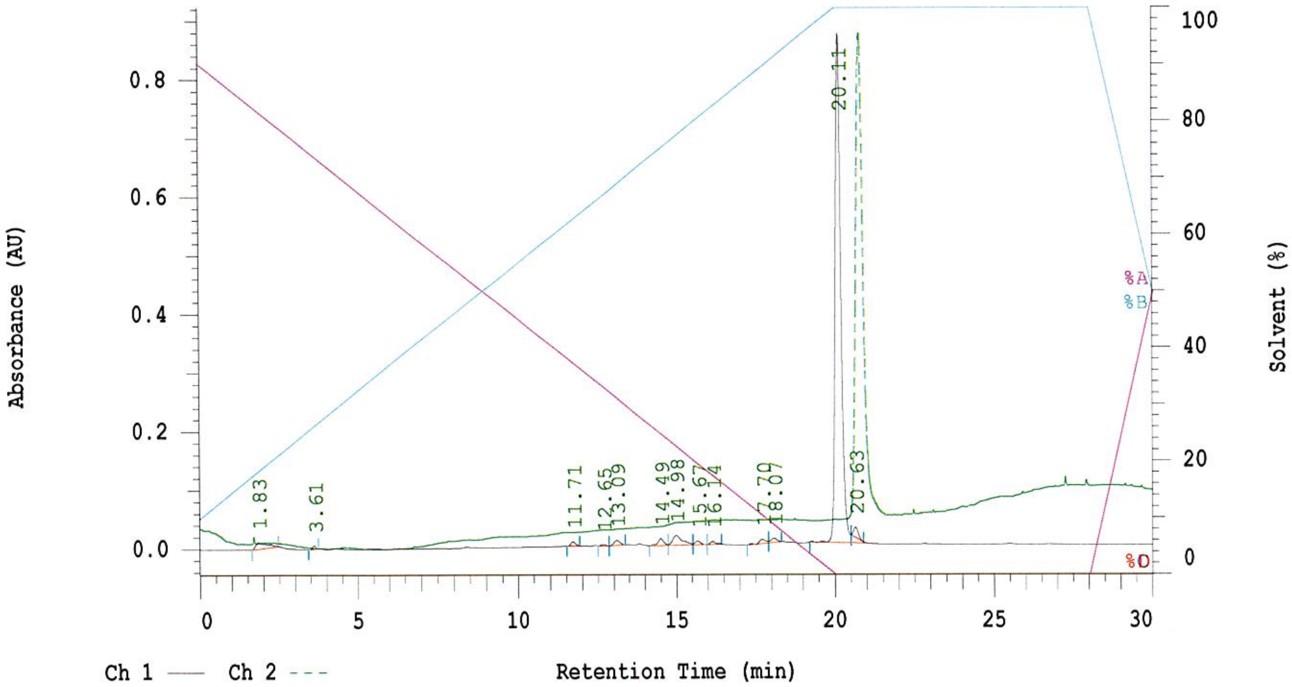

Acquisition Method: NOautosampler_30min_wELSD

Column Type: Vydac C18

Pump A Solvent A: H2O/5% ACN   Pump A Solvent B: ACN

Method Description:

Figure S4. High resolution DART positive ion Mass spectrum of Ilicicolin H used in the study

Elemental Compositions

D:\msAxel@LP Data\Amy data\Samples\Hb-322Calibrated.txt

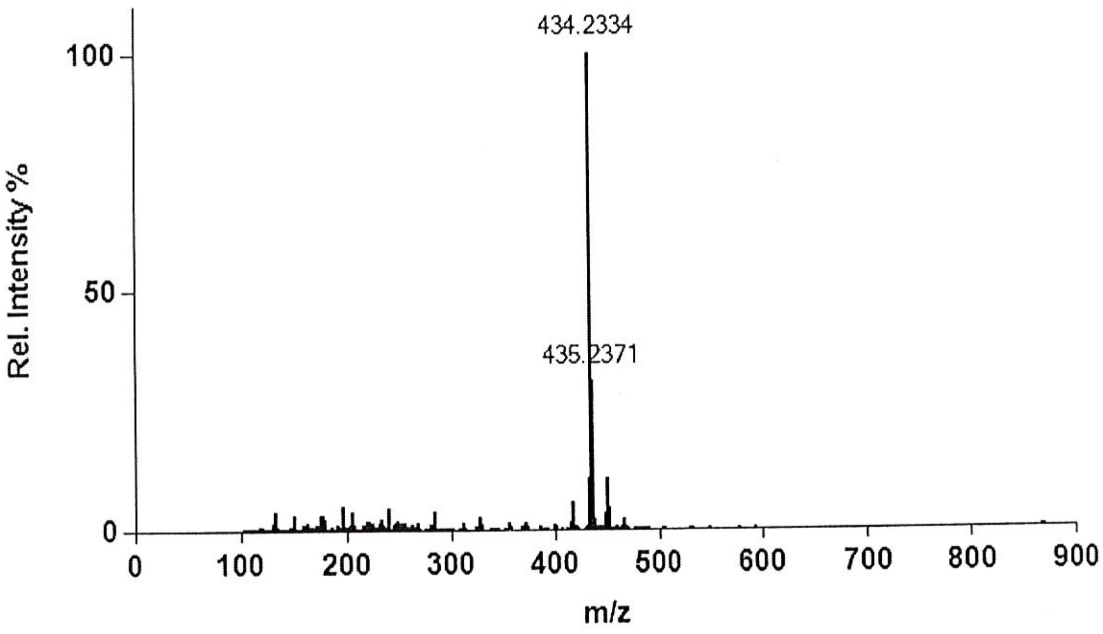

Mass Spectrum

```
Elementa l Compositions
Element  limits: C 0/28 H 0/33 O 0/5 N 0/1 Na 0/1
Tolerance: 10 mmuEven or odd electron ion or both: Even
Electron correction: None.Charges: 1
Minimum unsaturation: -1Maximum unsaturation: 100
```

Calc. m/z	Abund %	mmu	DBE	Composition
434.230728	100.000	-2.64	9.5	C25H33O4N1Na1
434.233133	100.000	-0.23	12.5	C27H32O4N1

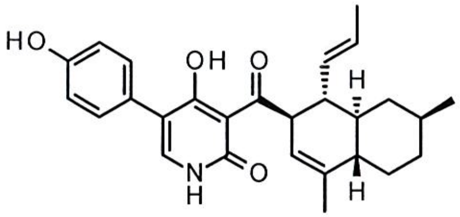

Ilicicolin H

Formula Weight : 433.54(2)
Formula : $C_{27}H_{31}NO_4$

Figure S5. HPLC chromatogram with PDA and ELSD detection of Tanzawaic Acid A

D-2000: JennSandle2017-2 Series: 0261 Report: original System: HPLC 1
018

D-2000 Elite HPLC System Manager Report

Analyzed: 09/13/2018 11:39 AM Reported: 09/13/2018 12:16 PM
Sample Name: HB-327
Sample Description: 1mg/ml

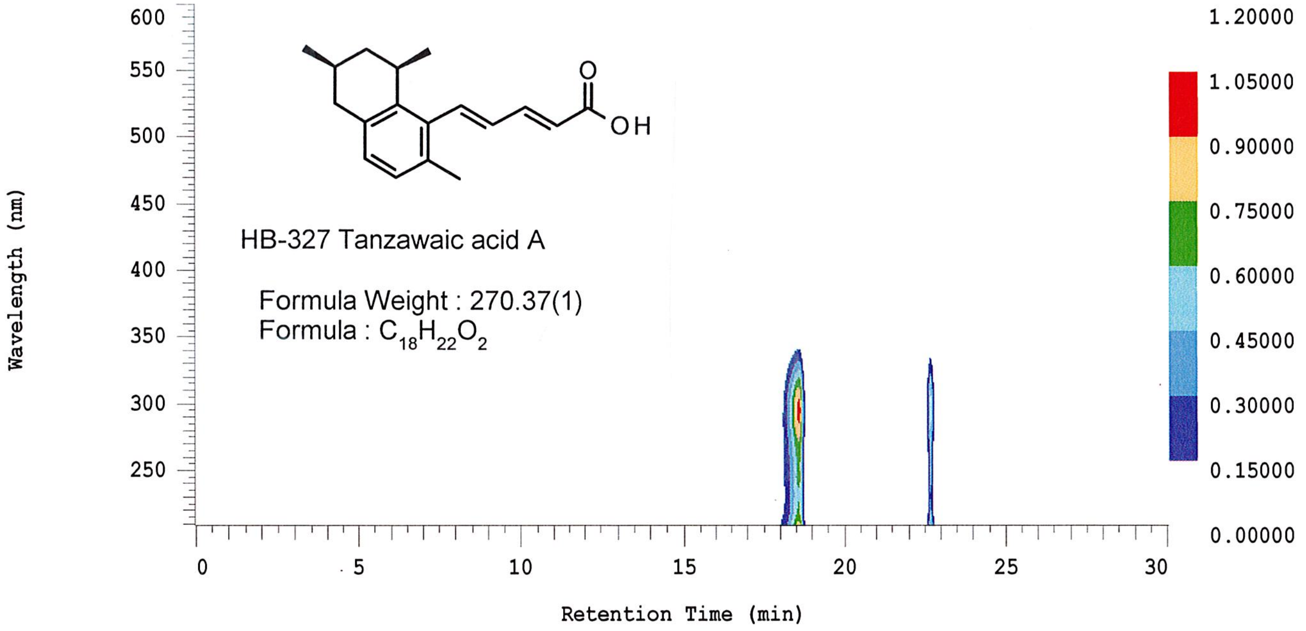

Channel 1 Chrom Type: Fixed WL Chromatogram, 230 nm

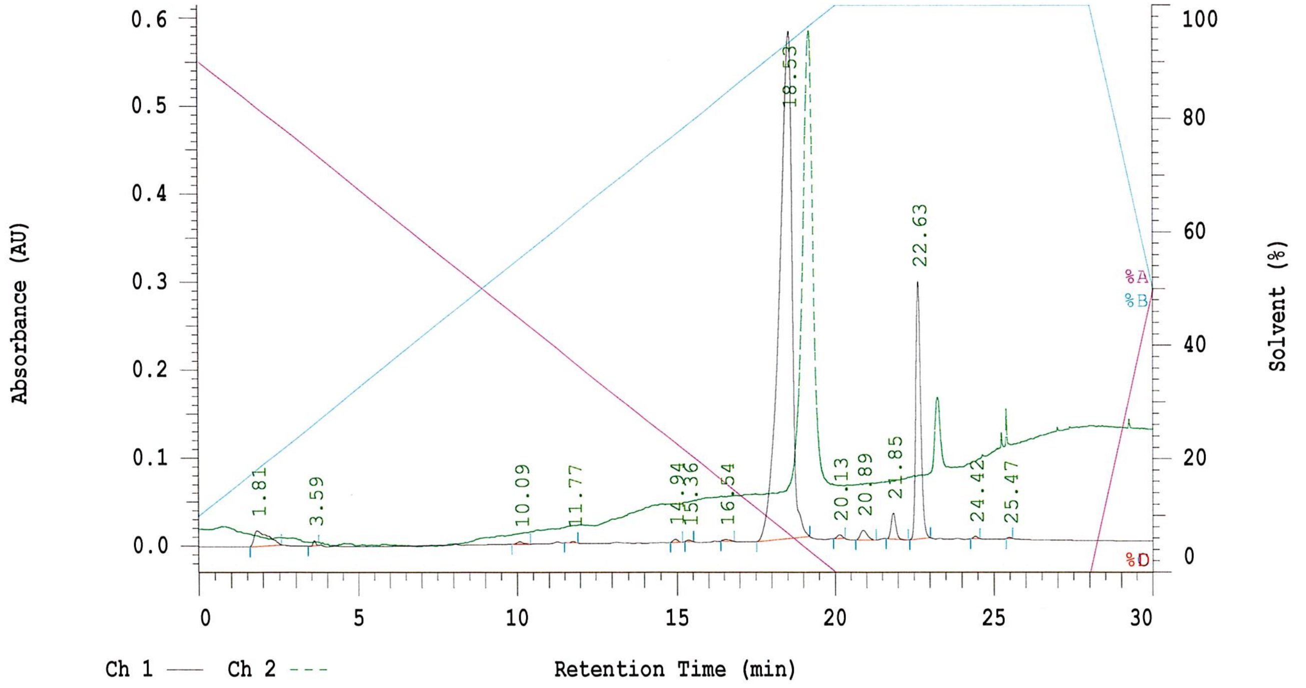

Acquisition Method: NOautosampler_30min_wELSD
Column Type: Vydac C18
Pump A Solvent A: H2O/5% ACN
Pump A Solvent B: ACN
Method Description:

Figure S6. High resolution DART positive ion Mass spectrum of Tanzawaic Acid A used in the study

Elemental Compositions

D:\msAxel@LP Data\Amy data\Samples\HB-327_tanzawaic Acid.txt

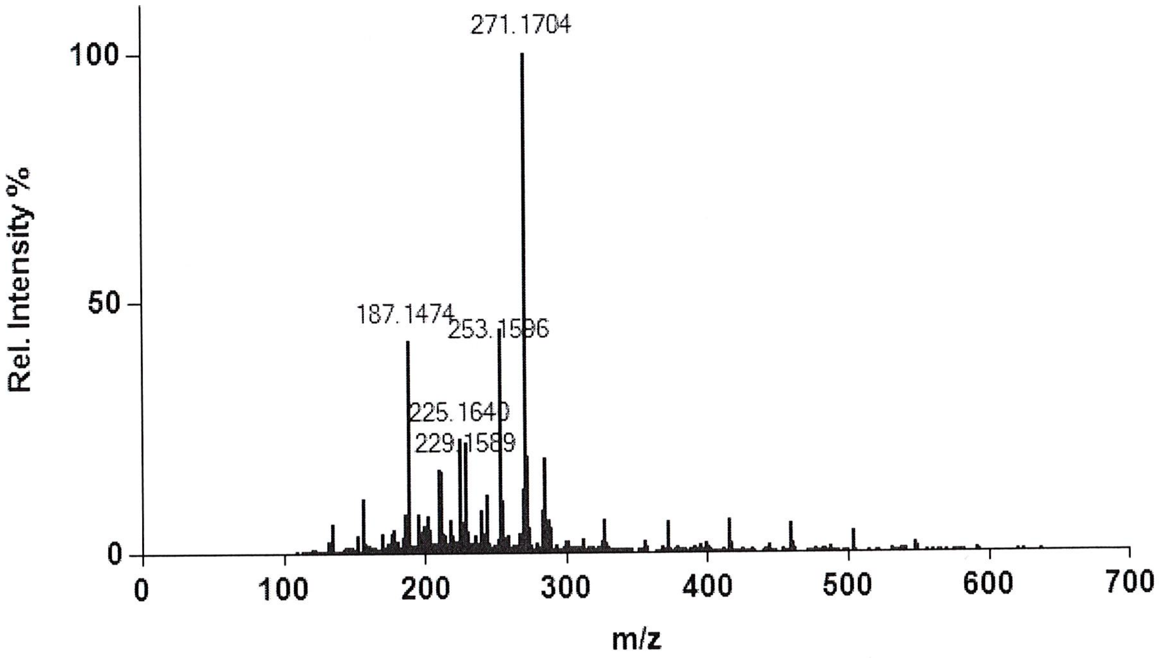

Mass Spectrum

Elemental Compositions
Element Limits: C 0/18 H 0/24 O 0/2 N 0/1 Na 0/1
Tolerance: 10 mmuEven or odd electron ion or both: Even
Electron correction: None.Charges: 1
Minimum unsaturation: -1Maximum unsaturation: 100

Calc. m/z	Abund %	mmu	DBE	Composition
271.167400	100.000	-2.98	4.5	C16H24O2Na1
271.169805	100.000	-0.57	7.5	C18H23O2

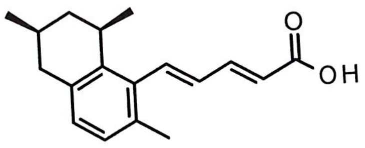

HB-327 Tanzawaic acid A

Formula Weight : 270.37(1)
Formula : $C_{18}H_{22}O_2$

Figure S7. HPLC chromatogram with PDA and ELSD detection of p-hydroyphenopyrrozin

D-2000: JennSandle2017-2 Series: 0262 Report: original System: HPLC 1
018

D-2000 Elite HPLC System Manager Report

Analyzed: 09/13/2018 12:16 PM Reported: 09/13/2018 12:49 PM
Sample Name: HB-331
Sample Description: 1mg/ml

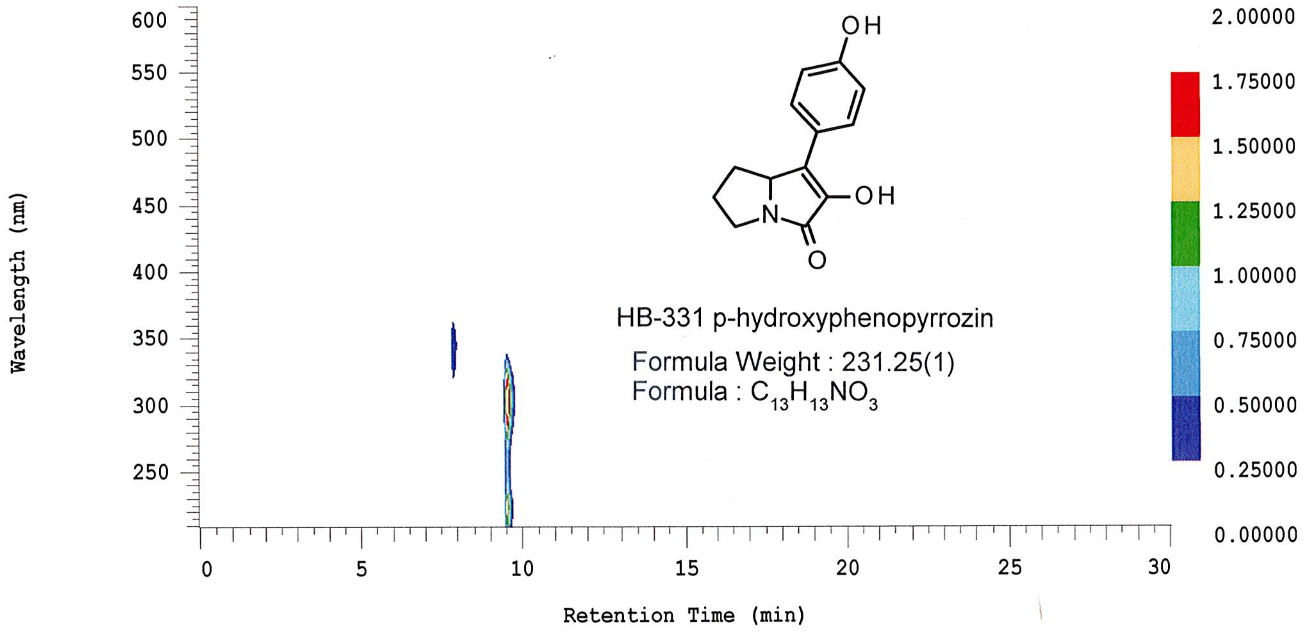

Channel 1 Chrom Type: Fixed WL Chromatogram, 230 nm

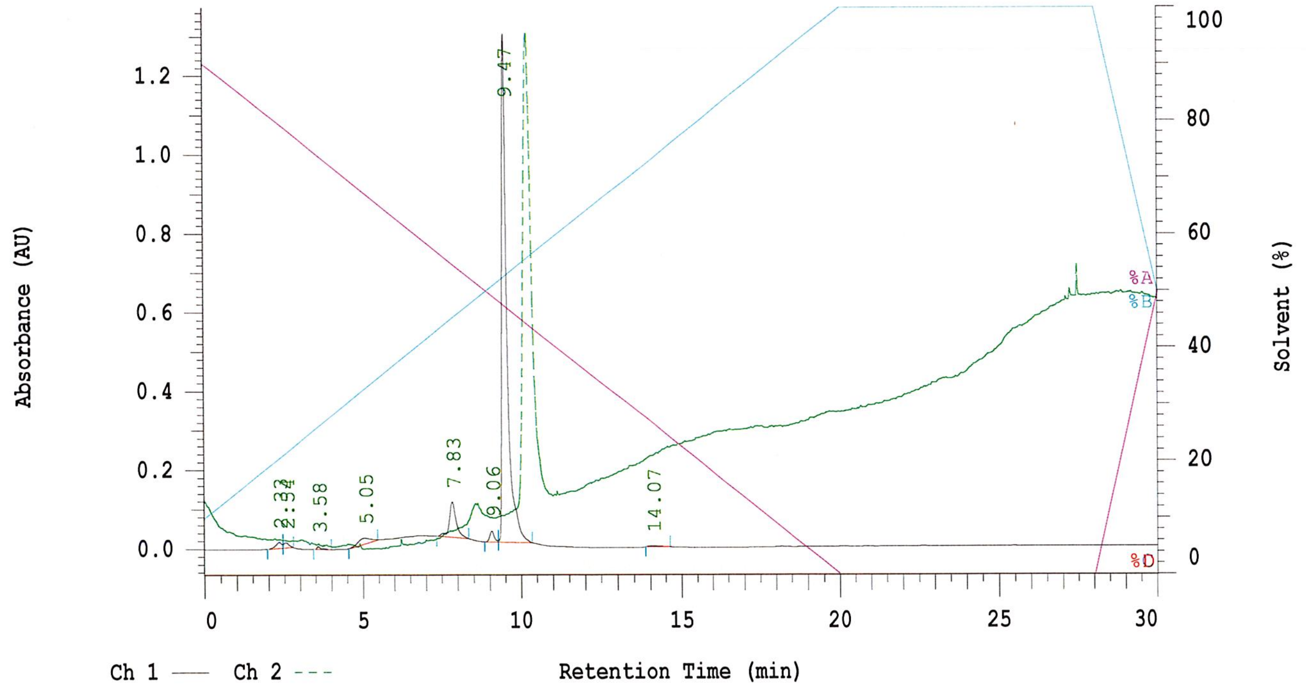

Acquisition Method: NOautosampler_30min_wELSD
Column Type: Vydac C18
Pump A Solvent A: H2O/5% ACN
Pump A Solvent B: ACN
Method Description:

Figure S8. High resolution DART Mass spectrum of p-hydroxyphenopyrrozin used in the study

Elemental Compositions

D:\msAxel@LP Data\Amy data\Samples\HB-331_tetrahydropyrrolin-3-one.txt

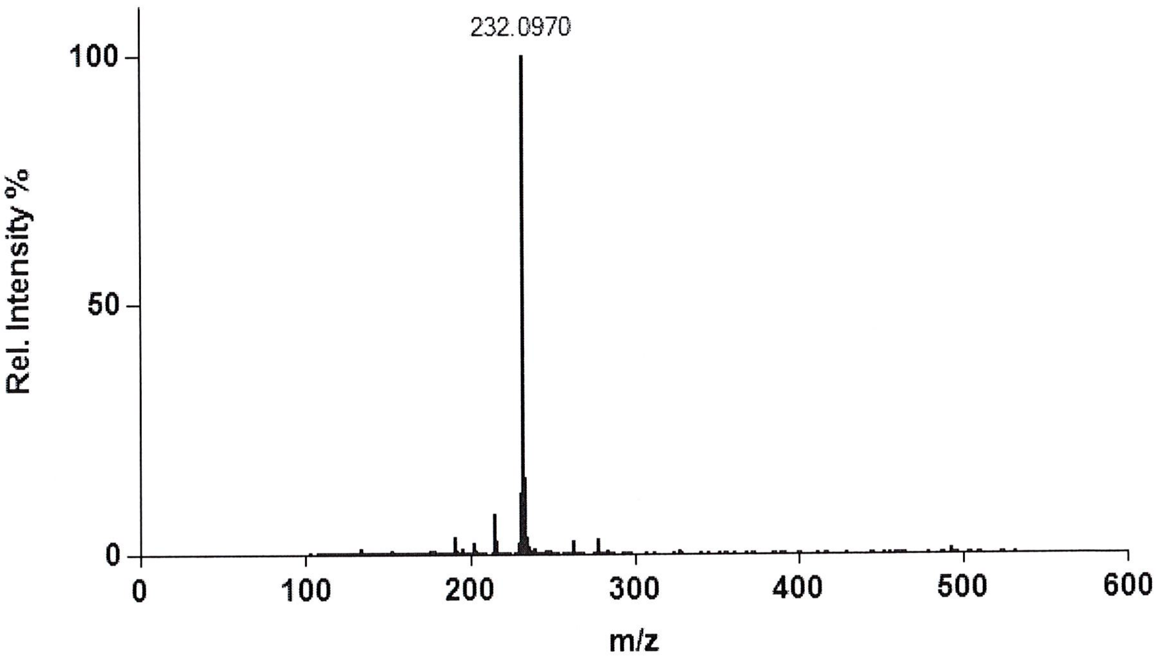

Mass Spectrum

Elemental Compositions
Element Limits: C 0/13 H 0/14 O 0/3 N 0/1 Na 0/1
Tolerance: 10 mmuEven or odd electron ion or both: Even
Electron correction: None.Charges: 1
Minimum unsaturation: -1Maximum unsaturation: 100

Calc. m/z	Abund %	mmu	DBE	Composition
232.097368	100.000	0.41	7.5	C13H14O3N1

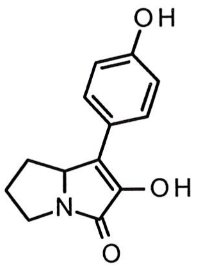

HB-331 p-hydroxyphenopyrrozin

Formula Weight : 231.25(1)
Formula : $C_{13}H_{13}NO_3$

Figure S9.

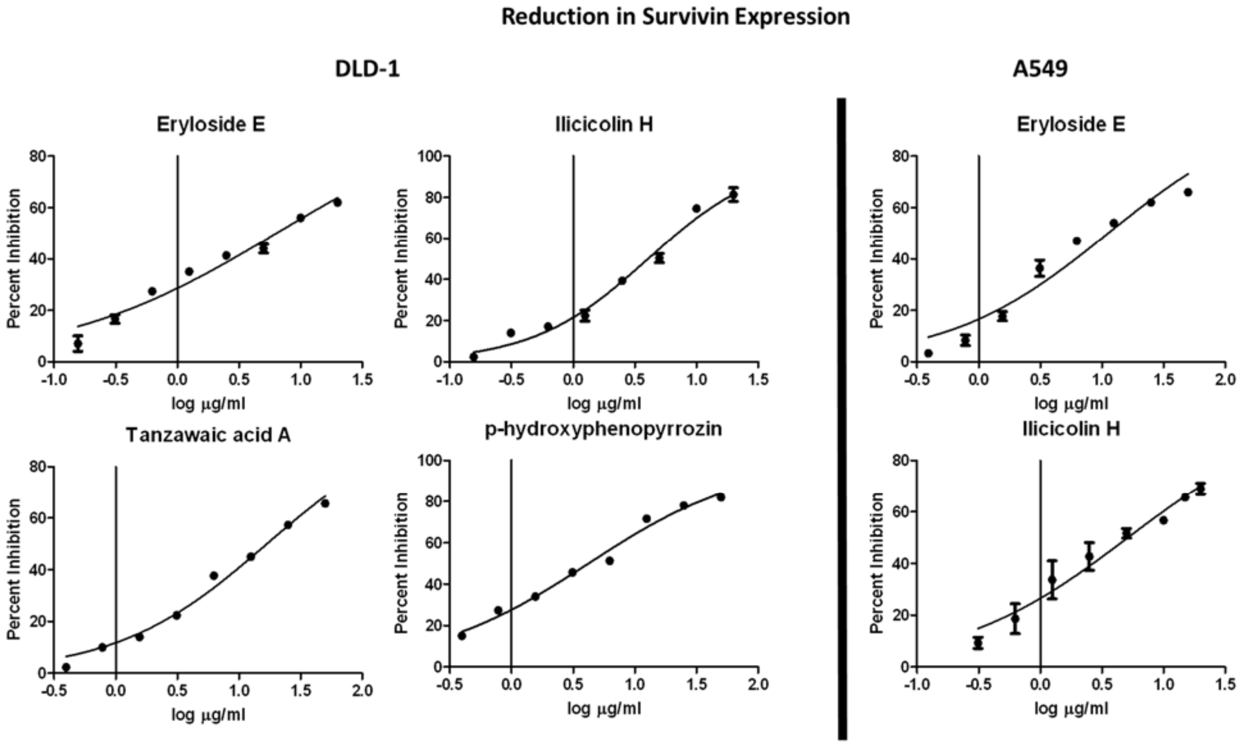

EC50 Graphs for the Reduction in Survivin Expression. Serial dilutions ranging from 20 to 0.04 µg/mL marine compounds were tested in the screening assay. Survivin expression levels were normalized to methanol (vehicle control) to express them as a percentage and subjected to a non-linear regression curve fit analysis using GraphPad Prism. The graphs show the average of 3 experiments ± standard deviation.

Figure S10.

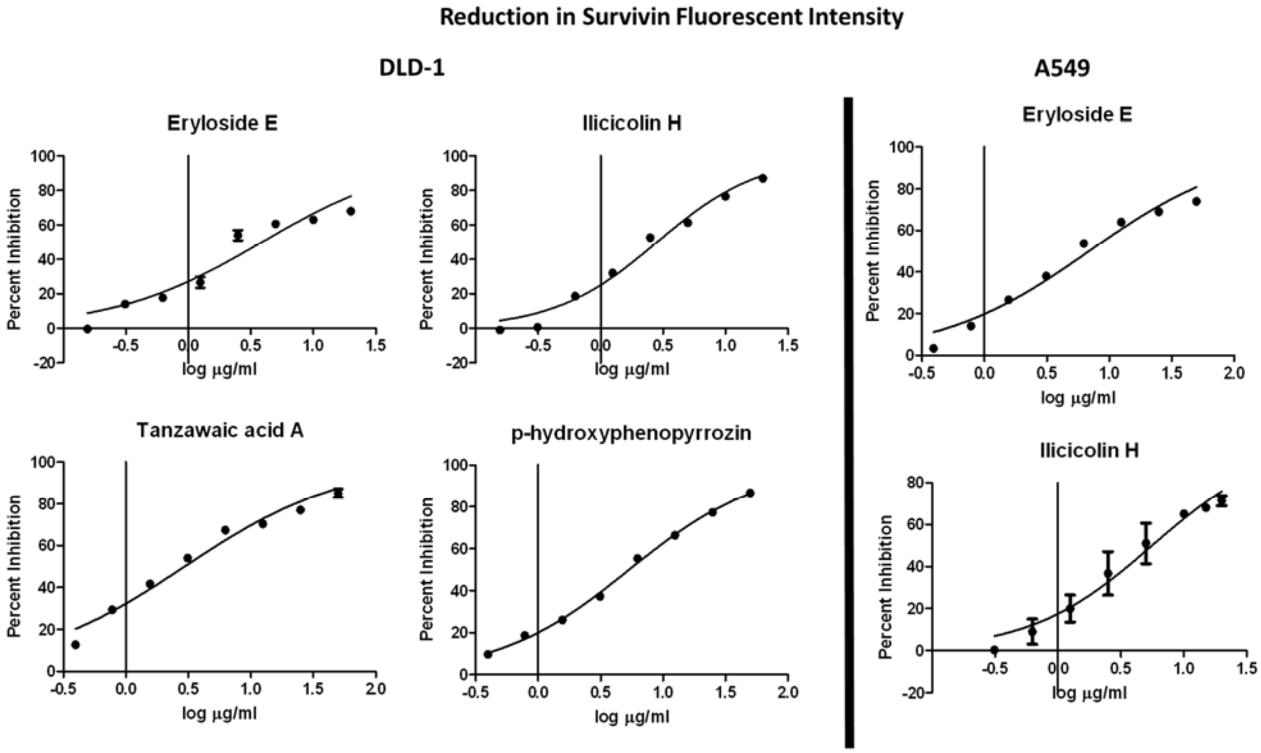

EC50 Graphs for the Reduction in Survivin Integrated Fluorescence Intensity. Serial dilutions ranging from 20 to 0.04 µg/mL marine compounds were tested in the screening assay. The integrated fluorescent intensity values for each concentration were normalized to methanol (vehicle control) to express them as a percentage and the values were subjected to a non-linear regression curve fit analysis using GraphPad Prism. The graphs show the average of 3 experiments ± standard deviation.

Figure S11.

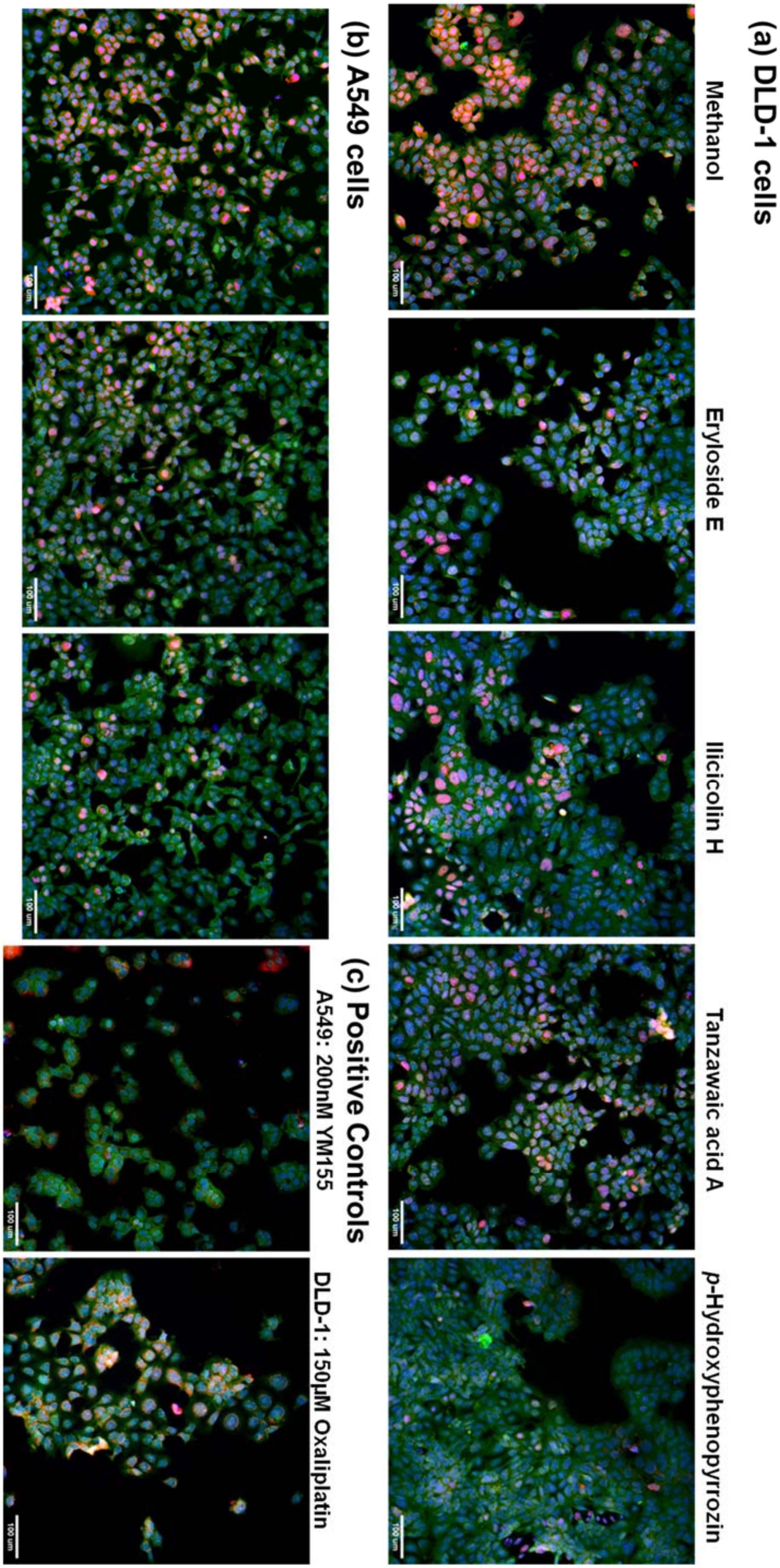

(a) DLD-1 cells
Methanol
Erylosside E
Ilicicolin H
Tanzawaic acid A
p-Hydroxyphenopyrrozin
(b) A549 cells
(c) Positive Controls
A549: 200nM YM155
DLD-1: 150μM Oxaliplatin
100 um

Figure S12.

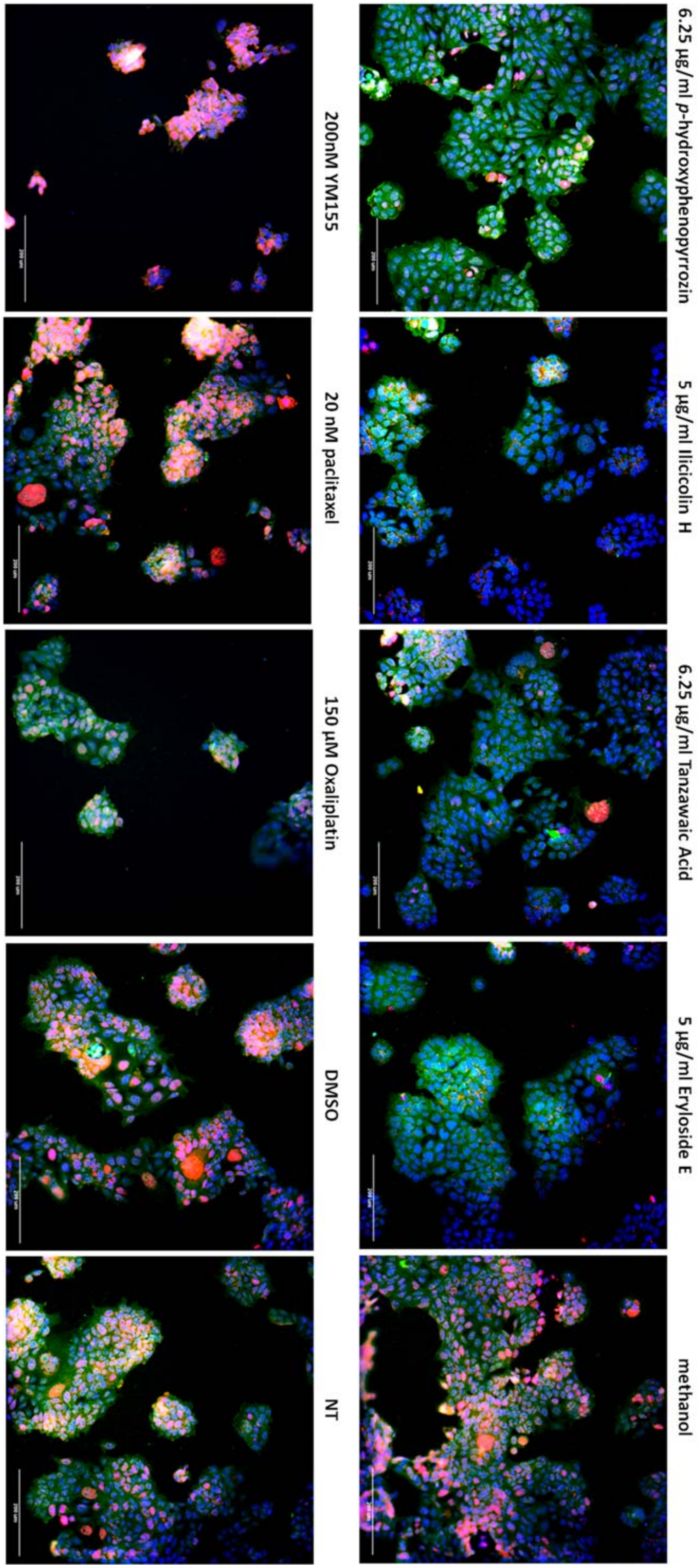
6.25 µg/ml *p*-hydroxyphenopyrrozin
5 µg/ml Ilicicolin H
6.25 µg/ml Tanzawaic Acid
5 µg/ml Eryloside E
methanol
200nM YM155
20 nM paclitaxel
150 µM Oxaliplatin
DMSO
NT
